# The Effect of Three Different Meditation Exercises on Hypertension: A Network Meta-Analysis

**DOI:** 10.1155/2017/9784271

**Published:** 2017-04-26

**Authors:** Hongchang Yang, Xueping Wu, Min Wang

**Affiliations:** ^1^Physical Education Department, Hohai University, Nanjing 210098, China; ^2^Shanghai Key Lab of Human Performance, Shanghai University of Sport, Shanghai 200438, China; ^3^School of Physical Education and Coaching, Shanghai University of Sport, Shanghai 200438, China; ^4^Scientific Research Department Academy of Science, Shanghai University of Sport, Shanghai 200438, China; ^5^Physical Education Department, Shanghai University of Finance and Economics, Shanghai 200433, China; ^6^School of Kinesiology, Shanghai University of Sport, Shanghai 200438, China

## Abstract

We aimed to use the pairwise and network meta-analysis to estimate the effects of different meditation exercises on the control of systolic blood pressure (SBP) and diastolic blood pressure (DBP). Randomized controlled trials (RCTs) were retrieved from PubMed and Embase up to June 2016, which are published in English and reported on meditation exercise for hypertensive patients. Risks of bias assessment of the included studies were assessed by Cochrane Collaboration Recommendations and network meta-analysis was performed by ADDIS. Mean difference (MD) and its 95% confidence interval (CI) were used as the effect size. A number of 19 RCTs were included in this study. Results of pairwise comparisons indicated that meditation exercise could significantly decrease the SBP and DBP, compared with other interventions (MD = −7.10, 95% CI: −10.82 to −3.39; MD = −4.02, 95% CI: −6.12 to −1.92). With good consistence and convergence, network meta-analysis showed that there were no significant differences between meditation and other interventions on SBP. For DBP, Qigong was significantly lower than “no intervention” (MD = −11.73, 95% CI: −19.85 to −3.69). Qigong may be the optimal exercise way in lowering SBP and DBP of hypertensive patients, but a detailed long-term clinical research should be needed in the future.

## 1. Introduction

Hypertension is one of the most common cardiovascular diseases worldwide with an increasing incidence among adolescents and adults. Increased systemic artery pressure is the major clinical manifestation of this disease. Hypertension is a risk factor for stroke, coronary heart disease, heart failure, renal insufficiency, and failure [1]. It is estimated that about 23.3 million deaths will occur in 2030, and about 80% of them are in low- and middle-income countries [2]. Despite the advanced antihypertensive medications and the increasing health care expenses, there are still two-thirds of hypertensive patients uncontrolled. Due to the above reasons, nonpharmacologic approaches, including exercise, physical activity, and life style modifications, have been recommended for the management of hypertension by the Joint National Committee [3].

Qigong is an ancient Chinese movement for people to improve their mind status. Qigong consists of series of exercises, such as meditation, breathing, rhythmical movements, and focus of intention. As its definition depicted, Qi is an important energy of the body and gong is the exercise that will promote Qi through the body so that the body can heal itself [1]. Previous studies have demonstrated that Qigong is beneficial in lowering hypertension as well as improving life qualities [4].

Similarly as Qigong, Tai Chi (also known as Shadow Boxing, Taiji, Tai Chi Chuan, or Tai Chi Quan) is another traditional Chinese exercise, which is performed dominantly by the elders to enhance body balance and awareness [5]. Since the 1980s, many of the scientific studies in both Chinese and English have reported Tai Chi is able to relieve some chronic syndromes, such as heart failure, rheumatoid arthritis, and human immunodeficiency related diseases [6, 7]. With the prevalence of meditation exercises, research and application of Tai Chi for hypertensive patients are also increased.

Yoga is a part of India traditional spiritual practice for individual to achieve the union of spirit, mind, and body. Despite its origins, Yoga has become a prevalent movement for mental and physical relaxing and a complementary method for chronic diseases control [2]. As a combination therapy, Yoga is also recommended to reduce the risk of cardiovascular causative factors such as hypertension [8], depression [9], and stress [10].

Although many articles have reported that meditation exercises, such as Qigong, Yoga, and Tai Chi, could effectively reduce the blood pressure and the effectiveness of them has been estimated by meta-analysis or summarized in a systematic review, the comparisons only focused on two of the interventions such as Yoga and care and Yoga and no active intervention [11, 12]; the simultaneous comparison among multiple meditation approaches is rarely reported. Therefore, in the current study, a network meta-analysis was conducted to comprehensively evaluate the effect of three meditation exercises including Qigong, Tai Chi, and Yoga on blood pressure reduction, so that an optimal strategy or some useful information can be obtained for the hypertension control in clinical treatment.

## 2. Methods

### 2.1. Literature Selection

Literatures were searched from electronic databases of PubMed (http://www.ncbi.nlm.nih.gov/pubmed/) and Embase (http://www.embase.com/) from their inception to June 2016 with English publications reported on the association between exercise and hypertension. The search strategy was set as the combinations of the following terms: hypertension (OR “high blood pressure” OR “Blood pressure”) AND Qigong (OR “Qi-gong” OR “chi-gong” OR “chi kung”) AND Yoga (OR “Yogic”) AND Tai Chi (OR “Taijiquan” OR “Shadow Boxing”).

### 2.2. Inclusion and Exclusion Criteria

Studies were included if they met the following criteria: (1) the articles investigated influence of meditation exercises such as Qigong, Yoga, and Tai Chi on the administrations of SBP and DBP in patients with hypertension; (2) the studies were randomized controlled trials (RCTs) and the treatment group were hypertensive patients intervened by meditation exercises such as Qigong, Yoga, or Tai Chi, while control group were hypertensive patients underwent walking, jogging, routine nursing, education, or “no intervention”; (3) articles could provide sufficient data to calculate the indexes of SBP and DBP after exercising by Qigong, Yoga, Tai Chi, or other interventions. However, studies were excluded if they were reviews, reports, comments, or negotiation letters.

### 2.3. Data Extraction and Risks of Bias Assessment

Data included in each eligible article was extracted by two independent authors. The extracted information included first authors' name, publication year, research country, and basic characteristics of participants such as gender, age, the interventions, and follow-up status. Risks of bias assessment were evaluated by the Cochrane Collaboration Recommendations assessment tools, which was recommended by the Cochrane Handbook [13]. Once any disagreement appeared during data extraction or assessment, the third investigator was required to discuss solutions.

### 2.4. Statistical Analysis

R 3.12 software (R Foundation for Statistical Computing, Beijing, China, meta package) was selected to perform the pairwise meta-analysis. Mean difference (MD) and its 95% confidence interval (CI) were used to present the effect size of the blood effect. Heterogeneity across trials was estimated by the *χ*^2^-based *Q* test [14] and *I*^2^ statistics, by which *p* value < 0.05 or *I*^2^ > 50% was considered to be heterogeneous and the random-effects model was chosen; otherwise (*p* value > 0.05 or *I*^2^ ≤ 50%), the fixed-effects model was selected [15]. Publication bias was examined by Egger's test [16].

Aggregate data drug information system (ADDIS, 1.16.5) was used for the network meta-analysis. This software was a nonprogramming software, which was based on Bayesian Framework and Markov Chain Monte Carlo (MCMC) theory and had a priori evaluation and processing for the research data [17, 18]. Similarly to pairwise comparison, MD with its 95% CI was also used as the effect size indicators for the measure of outcomes. Random-effects model was utilized to estimate the effect size in this study. As an alternative method for inconsistency assessment in network meta-analysis, node-splitting analysis was used to evaluate the consistency of data. When *p* value > 0.05, the consistency model was utilized; otherwise, the inconsistency model was selected [19]. Convergence of model was assessed by Brooks-Gelman-Rubin method, by which the major reference index was the potential scale reduction factor (PSRF). The more PSRF becomes close to 1, the better convergence presents. Normally, PSRF can be accepted less than 1.2 [20].

## 3. Results

### 3.1. Eligible Studies and Their Characteristics

**Figure 1:**
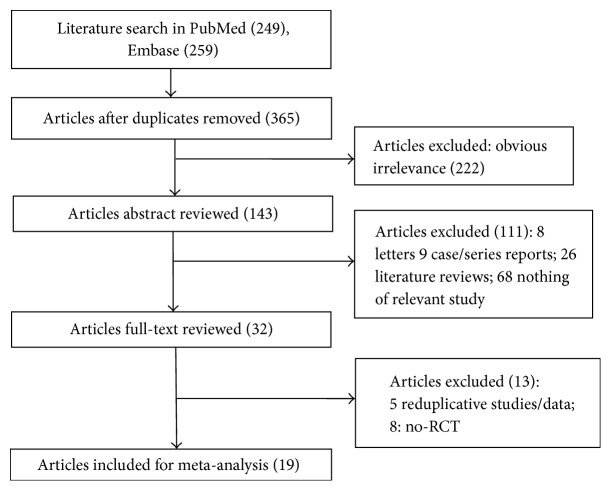
Flow chart of literature search and study selection.

A flowchart of literature searching and selection procedure was showed in Figure 1. According to the search strategy, a set of 508 papers were identified from PubMed (249) and Embase (259) databases. Of these, 143 were excluded due to duplication. After scanning the title or abstract, 222 papers were excluded from the remaining 365 researches on account of the contents obviously unrelated with our research. By further examination, 111 of the rest of the papers were excluded: letters (*n* = 8), case/series reports (*n* = 9), literature reviews (*n* = 26), and irrelevant studies (*n* = 68). Subsequently, the full-text of the remaining 32 articles was reviewed and 5 of them were duplicated and 8 were non-RCTs. Finally, a total of 19 eligible studies were included in this network meta-analysis [4, 21–38].

**Figure 2:**
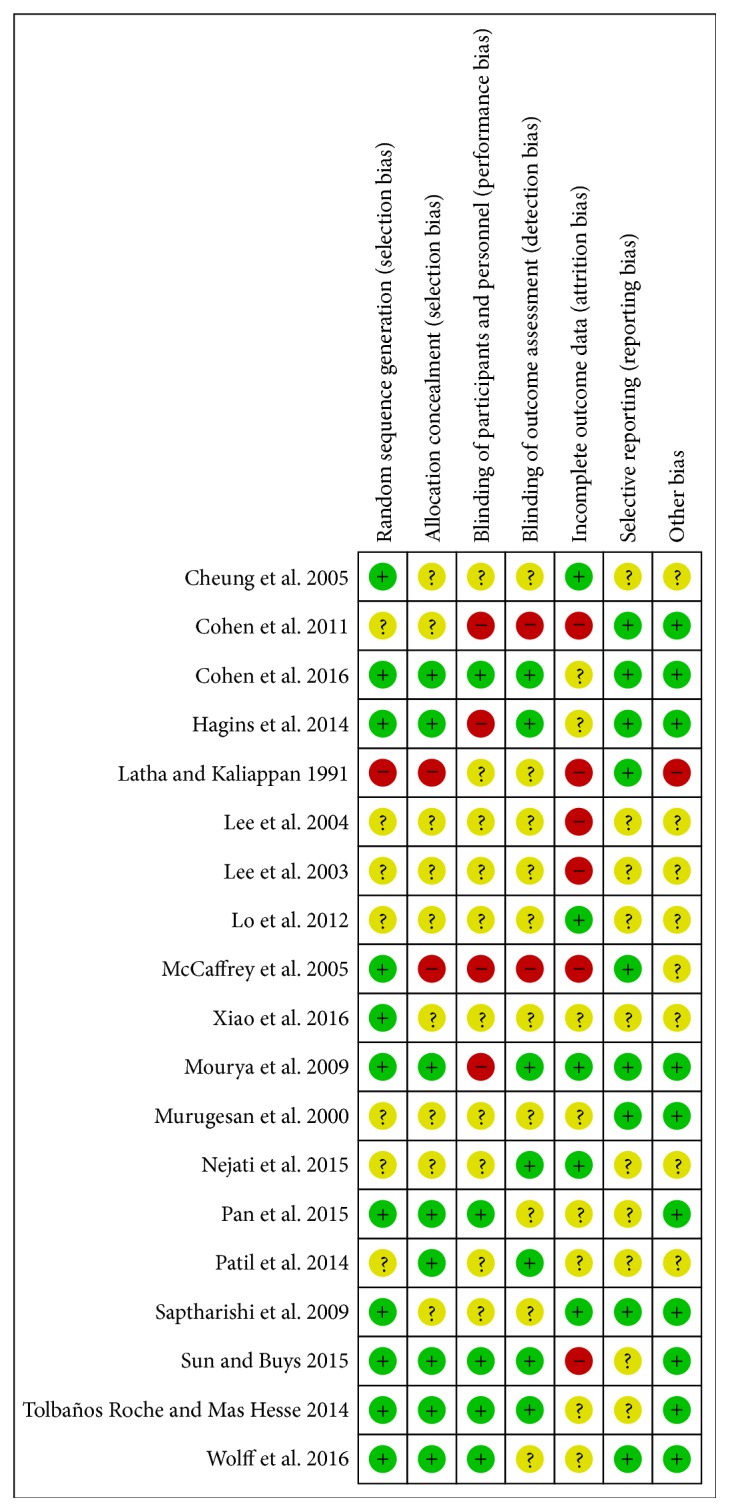
Risks of bias assessment.

The baseline characteristics of the included articles were summarized in Table 1. As revealed in this table, the identified 19 studies contained 1459 hypertensive patients, including 752 cases in the treatment group and 707 cases in the control group. The publication time of these studies ranged from 1991 to 2016, and most of them (18/19) were published after 2003. Participants mainly came from the following countries: Iran, America, Korea, Thailand, India, and China. All of the included patients were middle-aged, and there were no significant differences identified in the age and sex ratio terms. For the treatment group, Qigong, Yoga, and Tai Chi were the major interventions, while, in control group, patients just underwent normal exercise, routing nursing education, or “no intervention.” The follow-up period of both groups ranged from 8 weeks to 1 year. Risks of bias assessment indicated that the included studies had a good quality. However, several articles had relatively high risk of allocation concealment (selection bias), incomplete outcome data (attrition bias), blinding of participants and personnel (performance bias), and blinding of outcomes assessment (detection bias) (Figure 2).

### 3.2. Pairwise Meta-Analysis Results

**Figure 3:**
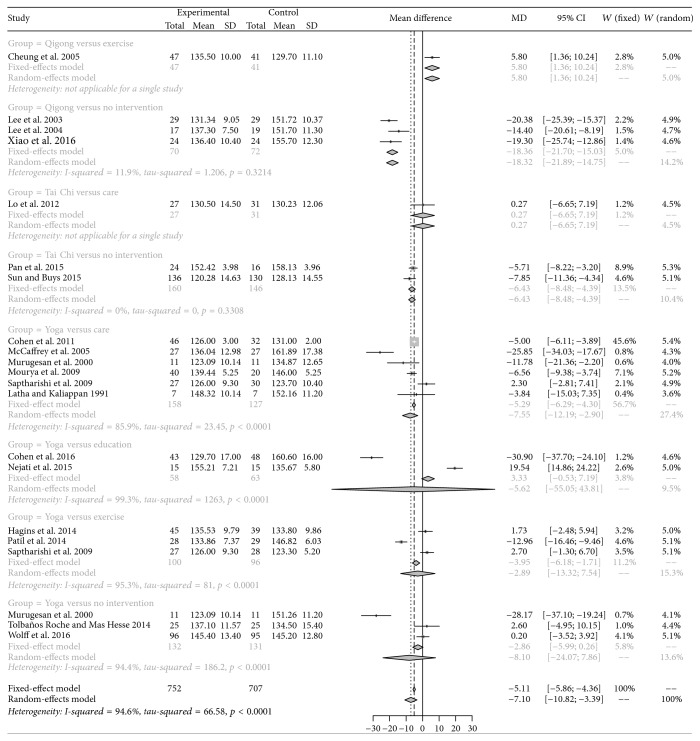
Pairwise comparison results of systolic blood pressure.

**Figure 4:**
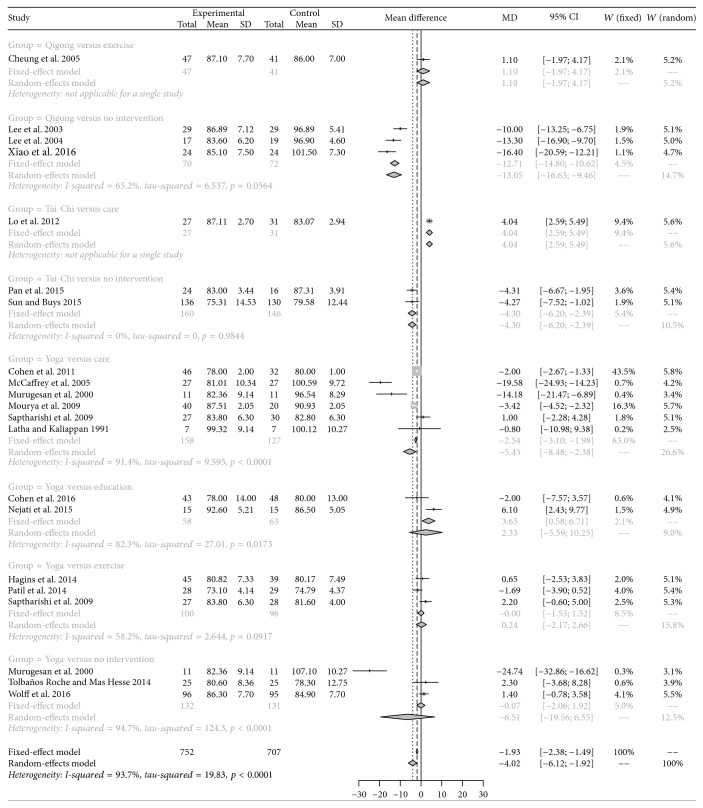
Pairwise comparison results of diastolic blood pressure.

According to significant heterogeneity (*p* < 0.05, *I*^2^ > 50%) estimated in systolic blood pressure (SBP) and diastolic blood pressure (DBP), random-effects model was selected to calculate the pooled results. As a result, medication exercises, including Qigong, Yoga, and Tai Chi, remarkably lowered the SBP and DBP of hypertensive patients compared with controls (MD = −7.10, 95% CI: −10.82 to −3.39; MD = −4.02, 95% CI: −6.12 to −1.92). Based on Egger's test results, there was no significant publication bias among studies regarding SBP (*t* = −0.669, *p* = 0.5115) and DBP (*t* = −1.2388, *p* = 0.2305), and these results reflected that results of our study had a relatively high reliability. Furthermore, subgroup analysis indicated that Tai Chi, Qigong, and Yoga also significantly decreased the SBPs and DBPs compared with other interventions, such as care and education (Figures 3 and 4).

### 3.3. Network Meta-Analysis

**Figure 5:**
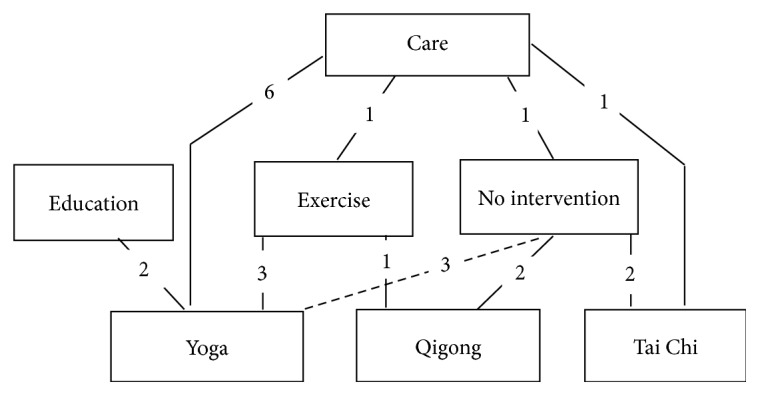
Network comparison of hypertension control.* Note*. The lines represent the comparison between two interventions and the numbers represent the numbers of the articles that provide these data.

ADDIS software was utilized to perform network meta-analysis, and the parameters were set as number of chains: 4, tuning iterations: 20000, simulation iterations: 50000, thinning interval: 10, inference samples: 10000, and variance scaling factor: 2.5. All the interventions were considered to construct a comprehensive network to show the investigations performed for both SBP and DBP (Figure 5). Because all the *p* values > 0.05 in node-splitting analysis (Tables 2 and 3) and PSRFs ranged from 1.00 to 1.01, good consistence of the included studies and better convergence of the model were obtained. Therefore, the consistency model is utilized for the subsequent network analysis.

The results of network meta-analysis were listed in Tables 4 and 5. In terms of SBP, Qigong showed a better outcome in SBP control but there were no significant differences detected compared with other interventions (Table 4), while, in terms of DBP, Qigong, education, and Yoga all presented promising reductions in DBP, but only Qigong showed a significant alteration compared with “no interventions” (MD = −11.73, 95% CI: −19.85 to −3.69, Table 5).

### 3.4. Rank Probability

**Figure 6:**
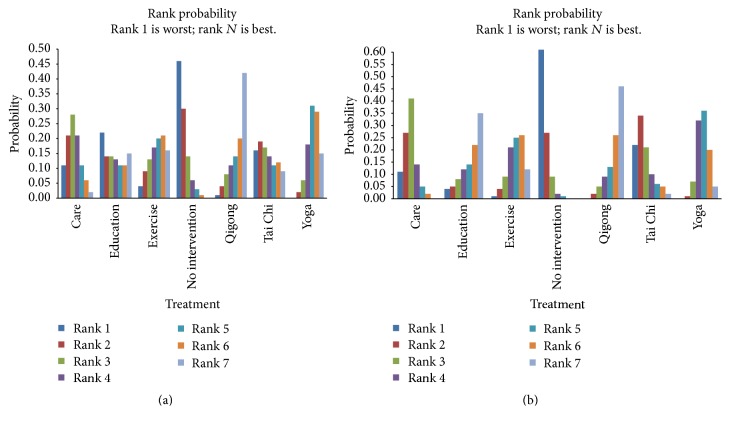
Rank probability of systolic blood pressure (a) and diastolic blood pressure (b).

The rank probability of hypertension was presented in Figure 6. For each intervention, the total rank probability was 1. A large portion of rank 1 represented a worse outcome, while a large portion of rank *N* represented a better effect outcome. In terms of SBP, both Qigong and Yoga had better outcomes than others, while “no intervention” had a worse outcome on SBP control, but the differences were not reflected compared with other interventions. In terms of DBP, both Qigong and Yoga had better outcomes and “no intervention” had a worse outcome on DBP control. Interestingly, only Qigong was significantly better than “no intervention” (MD = −11.73, 95% CI: −19.85 to −3.69).

## 4. Discussion

The effect of meditation on blood pressure control had been reported in many RCTs. However, the previous studies did not have a simultaneous systematic review of the relationships for all relevant evidences. For this article, 19 papers with 1459 patients were enrolled to illustrate the effects of different medication exercises on hypertension control. The pairwise meta-analysis in this study showed that Qigong, Yoga, and Tai Chi could significantly reduce the SBP and DBP of hypertensive patients, compared with no intervention, education, or exercise. However, results of network meta-analysis showed that only Qigong had a remarkable effect on lowering DBP, compared with “no intervention.”

Slow-breathing contributes to the decrease of heart rate by decreasing activities of both sympathetic and parasympathetic nervous systems, so that it can affect blood pressure [29]. Qigong, Tai Chi, and Yoga are the most common exercise types of meditation. Meditation is a common approach for anxiety reducing [39]. Lee has previously reported that Qigong can positively modulate blood pressure level and urinary catecholamine by stabilizing sympathetic nervous system which is responded to anxiety disorder [4]. Another research of Lee et al. also shows that Qigong has a significant benefit for SBP and DBP reduction after 8 weeks of exercise [25]. However, with small samples, a meta-analysis of Guo et al. has demonstrated that the self-practiced Qigong for less than 1 year has a better outcome in lowering hypertension compared with “no intervention,” but no superior outcomes were detected while compared with other interventions [1], which is also supported by Cheung et al. [21]. But in this research, results of pairwise analysis indicated that Qigong could significantly lower both SBP and DBP of hypertensive patients. The small discrepancy of Qigong's result between this and previous articles may be highly related to different sample sizes and follow-up times. For instance, in Guo's research, the sample size is small and the follow-up time is less than 1 year. However, in our study, the large sample size may reduce some influence caused by some potential bias risks despite the follow-up time which ranged from 8 weeks to 1 year. In addition, Qigong is usually performed in Asia, especially in China, and many of the researches were conducted among Chinese. In Guo's research, both English and Chinese published papers have been included, but in our study, only English written papers were included. Hence there will be a significant heterogeneity of the extracted SBP and DBP data in this article. Hagins et al. [40] and Tsai et al. [5] also have reported that Yoga and Tai Chi could significantly decrease the SBP and DBP of hypertensive patients than other interventions. In this research, results of the pairwise analysis indicated that Qigong, Tai Chi, and Yoga could significantly lower the SBP and DBP of hypertensive patients compared with “no intervention” as well as other exercises or education. As it is reported in many articles, mediation is a slow exercise and the benefits may need a longer time to realize than pharmacy treatment, so a long-term meditation effect analysis is needed in the future.

An important advantage of this study over previous researches is the ability to compare the different meditation exercises simultaneously and combine them into a comprehensive network; thus it can provide us with the best solution for hypertension control. Compared to the pairwise meta-analysis, this method not only can perform comparisons between each two of the included interventions, but also can simulate the real condition of pathogenesis. This means influence of the inventions could be estimated by series of comparisons. Based on this method, ADDIS were selected as the analytical tool to evaluate the influence of different inventions. Despite the good consistence and convergence of the test model, no significant differences of SBP and DBP were observed between different interventions, except the comparison between Qigong and “no intervention” on DBP according to network meta-analysis. Both SBP and DBP are the indicators of hypertension; why only DBP had the significant lower effect than “no intervention” is not clear. The same phenomenon is also identified in Veronique A's [41] research which focuses on exercise training for hypertension. According to his study, the significant reductions of SBP and DBP usually occur in male participants and prehypertension participants. Compared with this research, although a larger sample capacity was concerned, the gender and hypertension stages of participants were not taken into account, as well as the subgroup analysis. Although many studies have reported that Yoga has a beneficial effect on lowering SBP and DBP [34, 36], the network analysis result is already deficient. Till now, the pathogenesis of hypertension is still unclear and can only be described as a functional disorder disease which is highly related to people's life style, diet, weight, mood, exercise, smoking, and drinking [42, 43].

Several limitations of this network meta-analysis should be taken into consideration. First, due to the incomplete extracted data, several related criteria were not included, such as essential and primary hypertension, elders and adolescents, diabetes, and renal disease and cardiovascular disease, and the subgroup analysis was not allowed. Second, as we suggested in the context, Qigong, Yoga, and Tai Chi are most prevalent in Asian countries; therefore, several articles were published in Chinese or other non-English languages; but in order to improve quality of the included papers, published in English was used as a criterion in this study; hence, there may exist selection bias and some unknown impact on the final result. Third, the lowering ability of interventions may be exaggerated due to the unclosed circle data and fewer included papers. Last but not least, despite the fact that ADDIS has a simple operation, the constraint programming property may have a conservative effect on the final result.

## 5. Conclusion

In conclusion, results of the network meta-analysis suggest that Qigong may be a potential exercise pattern for hypertension control. Because Qigong is a chronic exercise and the outcome of it also comes slowly, therefore, this result still needed to be further verified by more eligible RCTs with large sample size and long-term clinical researches, as well as detailed subgroup analyses.

## Figures and Tables

**Table 1:** General characteristics of the included articles.

Author	Year	Location	M/F		Age, y		Treatment group	Control group	Follow-up
			T	C	T	C			
Lee et al.	2003	Korea	10/19	13/16	55.8 ± 6.3	57.1 ± 7.6	Shuxinpingxue gong 30 min/d	Wait-list control	10 wk
Lee et al.	2004	Korea	8/9	6/13	52.6 ± 5.1	54.3 ± 5.5	Shuxinpingxue gong 30 min/d	Wait-list control	8 wk
Cheung et al.	2005	China	21/26	16/25	57.2 ± 9.5	51.2 ± 7.4	Guolin qigong 60 min in the morning and 15 min in the evening/d	Conventional exercise: 60 min in the morning and 15 min in the evening/d	16 wk
Xiao et al.	2016	China	NA	NA	65.6 ± 7.8		NA	Wait-list control	6 ms
Cohen et al.	2011	USA	23/23	16/16	48.2 ± 1.6	48.3 ± 2.4	Iyengar Yoga; 2 × 70 min/week for 6 weeks + 1 × 70 min/week	Enhanced usual care; 4 × 60 min/week	12 wk
Cohen et al.	2016	USA	22/21	24/23	47.4 ±13	46.1 ± 13	Semiprivate structured classes, self-practice, community-structured classes	Blood Pressure Education Program	24 wk
Hagins et al.	2014	New Zealand	22/33	14/25	56.4 ± 9.78	52.45 ± 12.19	Modified Ashtanga Yoga; 2 × 55 min/week + 3 × 20 min/week home practice	Exercise, strengthening, stretching; 2 × 55 min/week + 3 × 20 min/week home practice	12 wk
Nejati et al.	2015	Iran	9/6	8/7	NA	NA	Yoga exercises	Mindfulness-based stress-reduction program	8 wk
McCaffrey et al.	2005	Thailand	10/17	9/18	Me: 56.7	Me: 56.2	Yoga; 3 × 63 min/week	Usual care	8 wk
Mourya et al.	2009	India	22/18	9/11	160.03 ± 5.21	158.10 ± 7.12	Yoga; 2 weeks to learn the breathing exercise technique + homework 2 × 15 min/day	Usual care	3 ms
Murugesan et al.	2000	India	NA	NA	35–65		Yoga; 6 × 120 min/week	(1) Usual care, f; (2) no treatment	11 wk
Patil et al.	2014	India	NA	NA	68.68 ± 4.97	69.17 ± 5.99	Yoga practice under the supervision of Yoga instructor for six days in a week for one hour daily	Practices for 15–20 min followed by walking for 35–40 min and rest for 5 min for six days in a week	3 ms
Saptharishi et al.	2009	India	15/10	39/17	22.5 ± 1.36	22.4 ± 1.3	Yoga; 5 × 30–45 min/week	(1) Usual care; (2) exercise 4 × 50–60 min/week	8 wk
Latha and Kaliappan	1991		NA	NA	NA	NA	Yoga; 17 × twice weekly	Usual care	6 ms
Tolbaños Roche and Mas Hesse	2014	Spain	8/17	10/15	57.70 ± 7.82	57.90 ± 8.67	Integrative Yoga Therapy	No treatment	3 ms
Wolff et al.	2016	Sweden	44/52	48/47	64.7 ± 9.2	64.8 ± 7.6	Yoga programme used in the study takes about 15 min to perform and incorporates the following exercises	No treatment	12 wk
Lo et al.	2012	Taiwan	33/25		58.47 ± 7.46		Yang-style Tai Chi exercise programme three times a week	Routine care with no Tai Chi exercise	8 wk
Pan et al.	2015	China	14/10	10/6	56.371 ± 3.95	56.88 ± 3.95	The 60 min exercise period began with a 10 min warm-up and ended with a 10 min wrap-up	No treatment	12 wk
Sun and Buys	2015	China	19/117	29/101	NA	NA	Participants were taught a variety of meditation techniques by an experienced trainer including breathing, balance, flexibility, concentration, and calming	No treatment	12 ms

T: treatment group; C: control group; wk: weeks; ms: months; na: not available; M: male; F: female; Me: mean age.

**Table 2:** Node-splitting analysis of systolic blood pressure.

Name	Direct effect	Indirect effect	Overall	*p* value
Care, exercise	−0.51 (−31.31, 29.68)	−5.89 (−27.80, 16.70)	−5.20 (−22.83, 11.64)	0.78
Care, no intervention	16.55 (−13.78, 46.37)	0.01 (−17.91, 17.27)	5.60 (−10.31, 21.37)	0.32
Care, Tai Chi	0.33 (−29.96, 30.67)	−1.72 (−30.28, 26.20)	−0.66 (−20.66, 19.75)	0.92
Care, Yoga	−8.36 (−20.05, 3.46)	4.48 (−18.76, 27.45)	−6.58 (−17.94, 4.58)	0.31
Exercise, Qigong	5.50 (−23.63, 34.09)	−12.09 (−40.33, 15.46)	−4.20 (−24.47, 16.31)	0.35
Exercise, Yoga	−3.08 (−20.02, 14.64)	1.39 (−24.30, 26.91)	−1.34 (−15.92, 13.49)	0.75
No intervention, Qigong	−18.17 (−35.20, −0.62)	0.04 (−36.64, 36.39)	−14.91 (−30.22, 0.25)	0.34
No intervention, Tai Chi	−6.67 (−28.30, 15.10)	−5.04 (−41.31, 30.83)	−6.29 (−23.92, 11.83)	0.93
No intervention, Yoga	−8.04 (−25.19, 9.46)	−22.58 (−44.52, −0.74)	−12.18 (−26.54, 2.32)	0.28

**Table 3:** Node-splitting analysis of diastolic blood pressure.

Name	Direct effect	Indirect effect	Overall	*p* value
Care, exercise	−1.38 (−17.16, 15.08)	−5.95 (−17.68, 5.80)	−5.00 (−14.10, 3.61)	0.62
Care, no intervention	11.98 (−4.20, 27.10)	−0.40 (−9.34, 8.21)	3.83 (−4.65, 12.47)	0.16
Care, Tai Chi	4.21 (−11.47, 19.85)	−1.92 (−16.38, 13.43)	1.12 (−8.93, 11.63)	0.55
Care, Yoga	−6.28 (−12.05, −0.83)	5.07 (−5.50, 15.87)	−4.47 (−10.68, 1.62)	0.06
Exercise, Qigong	1.09 (−14.76, 16.18)	−6.87 (−21.23, 8.51)	−2.94 (−13.17, 7.69)	0.43
Exercise, Yoga	0.24 (−8.81, 9.63)	0.42 (−13.45, 13.39)	0.58 (−7.17, 8.23)	0.98
No intervention, Qigong	−13.10 (−22.30, −4.07)	−5.37 (−25.48, 14.46)	−11.73 (−19.85, −3.69)	0.46
No intervention, Tai Chi	−4.32 (−15.58, 6.84)	1.67 (−16.81, 20.25)	−2.73 (−11.89, 6.53)	0.56
No intervention, Yoga	−5.80 (−14.95, 3.14)	−13.49 (−25.43, −2.34)	−8.31 (−15.91, −0.74)	0.25

**Table 4:** Network meta-analysis results of systolic blood pressure.

Care	−1.09 (−25.00, 22.75)	−5.20 (−22.83, 11.64)	5.60 (−10.31, 21.37)	−9.44 (−29.38, 11.38)	−0.66 (−20.66, 19.75)	−6.58 (−17.94, 4.58)
1.09 (−22.75, 25.00)	Education	−4.11 (−29.84, 21.84)	6.86 (−18.68, 32.39)	−8.04 (−36.95, 20.32)	0.59 (−29.23, 30.12)	−5.32 (−26.52, 15.87)
5.20 (−11.64, 22.83)	4.11 (−21.84, 29.84)	Exercise	10.85 (−7.61, 29.21)	−4.20 (−24.47, 16.31)	4.64 (−19.31, 28.84)	−1.34 (−15.92, 13.49)
−5.60 (−21.37, 10.31)	−6.86 (−32.39, 18.68)	−10.85 (−29.21, 7.61)	No intervention	−14.91 (−30.22, 0.25)	−6.29 (−23.92, 11.83)	−12.18 (−26.54, 2.32)
9.44 (−11.38, 29.38)	8.04 (−20.32, 36.95)	4.20 (−16.31, 24.47)	14.91 (−0.25, 30.22)	Qigong	8.67 (−13.94, 31.54)	2.79 (−16.31, 21.92)
0.66 (−19.75, 20.66)	−0.59 (−30.12, 29.23)	−4.64 (−28.84, 19.31)	6.29 (−11.83, 23.92)	−8.67 (−31.54, 13.94)	Tai Chi	−5.77 (−26.17, 14.55)
6.58 (−4.58, 17.94)	5.32 (−15.87, 26.52)	1.34 (−13.49, 15.92)	12.18 (−2.32, 26.54)	−2.79 (−21.92, 16.31)	5.77 (−14.55, 26.17)	Yoga

**Table 5:** Network meta-analysis results of diastolic blood pressure.

Care	−6.71 (−19.46, 5.97)	−5.00 (−14.10, 3.61)	3.83 (−4.65, 12.47)	−7.96 (−18.65, 3.19)	1.12 (−8.93, 11.63)	−4.47 (−10.68, 1.62)
6.71 (−5.97, 19.46)	Education	1.64 (−11.93, 15.49)	10.45 (−3.07, 24.24)	−1.28 (−16.22, 14.14)	7.74 (−7.76, 23.16)	2.26 (−8.92, 13.51)
5.00 (−3.61, 14.10)	−1.64 (−15.49, 11.93)	Exercise	8.80 (−0.65, 18.73)	−2.94 (−13.17, 7.69)	6.06 (−5.70, 18.49)	0.58 (−7.17, 8.23)
−3.83 (−12.47, 4.65)	−10.45 (−24.24, 3.07)	−8.80 (−18.73, 0.65)	No intervention	−11.73 (−19.85, −3.69)	−2.73 (−11.89, 6.53)	−8.31 (−15.91, −0.74)
7.96 (−3.19, 18.65)	1.28 (−14.14, 16.22)	2.94 (−7.69, 13.17)	11.73 (3.69, 19.85)	Qigong	8.99 (−2.63, 21.06)	3.46 (−6.81, 13.24)
−1.12 (−11.63, 8.93)	−7.74 (−23.16, 7.76)	−6.06 (−18.49, 5.70)	2.73 (−6.53, 11.89)	−8.99 (−21.06, 2.63)	Tai Chi	−5.55 (−16.20, 4.68)
4.47 (−1.62, 10.68)	−2.26 (−13.51, 8.92)	−0.58 (−8.23, 7.17)	8.31 (0.74, 15.91)	−3.46 (−13.24, 6.81)	5.55 (−4.68, 16.20)	Yoga

## Abbreviations

SBP: Systolic blood pressure
DBP: Diastolic blood pressure
RCTs: Randomized controlled trials
MD: Mean difference
CI: Confidence interval
ADDIS: Aggregate data drug information system
MCMC: Markov Chain Monte Carlo
PSRF: The potential scale reduction factor.
